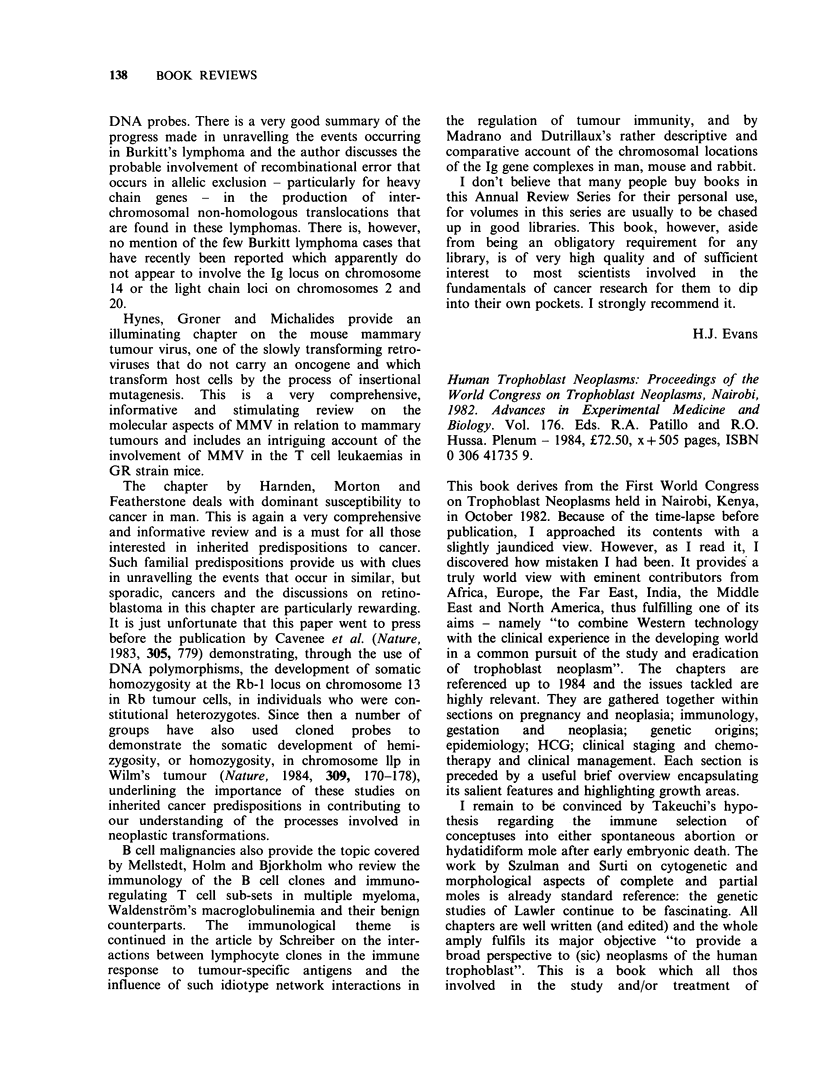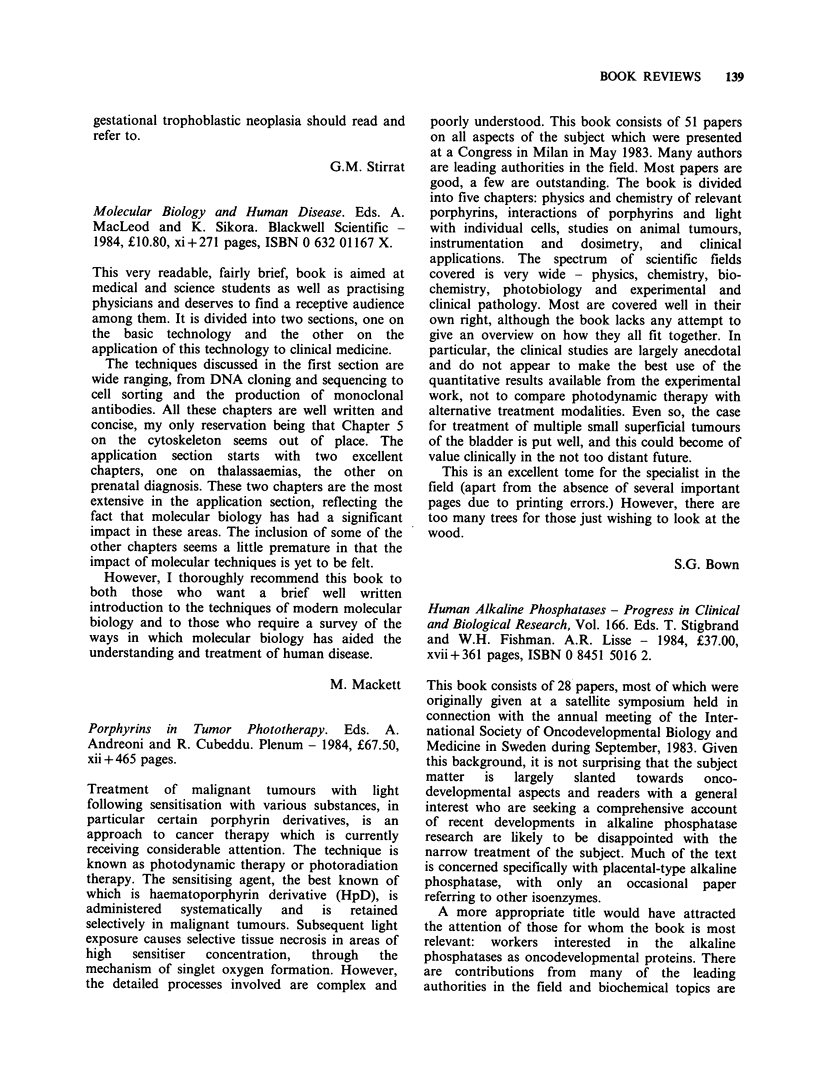# Human Trophoblast Neoplasms: Proceedings of the World Congress on Trophoblast Neoplasms, Nairobi, 1982. Advances in Experimental Medicine and Biology

**Published:** 1985-07

**Authors:** G.M. Stirrat


*Human Trophoblast Neoplasms: Proceedings of the World Congress on Trophoblast Neoplasms, Nairobi, 1982. Advances in Experimental Medicine and Biology*. Vol. 176. Eds. R.A. Patillo and R.O. Hussa. Plenum – 1984, £72.50, x + 505 pages, ISBN 0 306 41735 9.

This book derives from the First World Congress on Trophoblast Neoplasms held in Nairobi, Kenya, in October 1982. Because of the time-lapse before publication, I approached its contents with a slightly jaundiced view. However, as I read it, I discovered how mistaken I had been. It provides a truly world view with eminent contributors from Africa, Europe, the Far East, India, the Middle East and North America, thus fulfilling one of its aims – namely "to combine Western technology with the clinical experience in the developing world in a common pursuit of the study and eradication of trophoblast neoplasm". The chapters are referenced up to 1984 and the issues tackled are highly relevant. They are gathered together within sections on pregnancy and neoplasia; immunology, gestation and neoplasia; genetic origins; epidemiology; HCG; clinical staging and chemo-therapy and clinical management. Each section is preceded by a useful brief overview encapsulating its salient features and highlighting growth areas.

I remain to be convinced by Takeuchi's hypo-thesis regarding the immune selection of conceptuses into either spontaneous abortion or hydatidiform mole after early embryonic death. The work by Szulman and Surti on cytogenetic and morphological aspects of complete and partial moles is already standard reference: the genetic studies of Lawler continue to be fascinating. All chapters are well written (and edited) and the whole amply fulfils its major objective "to provide a broad perspective to (sic) neoplasms of the human trophoblast". This is a book which all thos involved in the study and/or treatment of gestational trophoblastic neoplasia should read and refer to.

G.M. Stirrat